# Exploration of the Anti-Inflammatory Drug Space Through Network Pharmacology: Applications for Drug Repurposing

**DOI:** 10.3389/fphys.2018.00151

**Published:** 2018-03-01

**Authors:** Guillermo de Anda-Jáuregui, Kai Guo, Brett A. McGregor, Junguk Hur

**Affiliations:** Department of Biomedical Sciences, School of Medicine & Health Sciences, University of North Dakota, Grand Forks, ND, United States

**Keywords:** anti-inflammatory drugs, network pharmacology, adverse drug reactions, pathways, systems pharmacology, drug repurposing

## Abstract

The quintessential biological response to disease is inflammation. It is a driver and an important element in a wide range of pathological states. Pharmacological management of inflammation is therefore central in the clinical setting. Anti-inflammatory drugs modulate specific molecules involved in the inflammatory response; these drugs are traditionally classified as steroidal and non-steroidal drugs. However, the effects of these drugs are rarely limited to their canonical targets, affecting other molecules and altering biological functions with system-wide effects that can lead to the emergence of secondary therapeutic applications or adverse drug reactions (ADRs). In this study, relationships among anti-inflammatory drugs, functional pathways, and ADRs were explored through network models. We integrated structural drug information, experimental anti-inflammatory drug perturbation gene expression profiles obtained from the Connectivity Map and Library of Integrated Network-Based Cellular Signatures, functional pathways in the Kyoto Encyclopedia of Genes and Genomes (KEGG) and Reactome databases, as well as adverse reaction information from the U.S. Food and Drug Administration (FDA) Adverse Event Reporting System (FAERS). The network models comprise nodes representing anti-inflammatory drugs, functional pathways, and adverse effects. We identified structural and gene perturbation similarities linking anti-inflammatory drugs. Functional pathways were connected to drugs by implementing Gene Set Enrichment Analysis (GSEA). Drugs and adverse effects were connected based on the proportional reporting ratio (PRR) of an adverse effect in response to a given drug. Through these network models, relationships among anti-inflammatory drugs, their functional effects at the pathway level, and their adverse effects were explored. These networks comprise 70 different anti-inflammatory drugs, 462 functional pathways, and 1,175 ADRs. Network-based properties, such as degree, clustering coefficient, and node strength, were used to identify new therapeutic applications within and beyond the anti-inflammatory context, as well as ADR risk for these drugs, helping to select better repurposing candidates. Based on these parameters, we identified naproxen, meloxicam, etodolac, tenoxicam, flufenamic acid, fenoprofen, and nabumetone as candidates for drug repurposing with lower ADR risk. This network-based analysis pipeline provides a novel way to explore the effects of drugs in a therapeutic space.

## Introduction

Inflammation is a complex phenomenon involving immune cell recruitment in response to harmful stimuli as a protective measure by the body. The recruitment process is achieved by a wide variety of cytokines released by resident immune cells that can be either pro- or anti-inflammatory. Inflammation begins as an acute response aimed toward clearance of stimuli as well as mediators in an effort to restore normal function. Inflammatory mediators involved in an acute response are often short-lived, leading to resolution of inflammation once the stimuli are cleared (Cotran et al., 2015). However, acute inflammation can shift to chronic inflammation if the stimuli are not removed. An immune response resulting in an inflamed condition is triggered by a wide variety of stimuli, such as pathogens, damaged cells, or irritants (Ferrero-Miliani et al., 2007). As such, inflammation is central to a myriad of pathological manifestations that result in a collection of complex and non-linear biological processes involved in an organism's response to stimuli (Vodovotz et al., 2010). Management of these processes with anti-inflammatory drugs is an important part of medical practice.

Even though interactions between molecules involved in the inflammatory response give way to a complex system (Vodovotz et al., 2008), most pharmacological drugs that deal with the response do so by modulating concrete molecular targets along the biochemical pathway of arachidonic acid to eicosanoids (Haeggström et al., 2010). Traditionally, two classes of anti-inflammatory drugs exist: corticosteroids and non-steroidal anti-inflammatory drugs (NSAIDS) (Agambar and Flower, 1990; Dinarello, 2010). The canonical targets through which these drugs exert anti-inflammatory effects are phospholipase A2 and cyclooxygenase (COX) 1 and 2 (Bozimowski, 2015).

However, anti-inflammatory drugs may have effects on molecules beyond these canonical targets (Barnes, 2006; Palayoor et al., 2012), and these off-site effects may have fortunate and unfortunate consequences. The effects of drugs on unintended targets can be the origin of adverse drug reactions (ADRs) and side effects (Rudmann, 2013); however, effects on alternative molecular and functional targets can lead to the *repurposing* of a drug, in which the drug is used in an alternative therapeutic application (Chartier et al., 2017). A successful pharmaceutical drug will strike an adequate balance between its therapeutic and unintended toxic effects. Drug repositioning can overcome limitations in development pipelines by dealing with drugs that have already been tested and marketed, reducing the risk of failure at the development stage. Anti-inflammatory drugs have been identified in drug repurposing studies (Strittmatter, 2014); however, there has been no systematic analysis of their functional and adverse effects.

Large-scale datasets related to drugs are common and contain information on chemical structures, adverse effects in the clinical setting, and system-wide effects from high-throughput drug screening projects. The magnitude of these datasets requires alternative analytical strategies from those of traditional pharmacological approaches, which can generate hypotheses and guide adequate experimental designs. In this context, network-based models can be useful (Hopkins, 2008).

A biological network is a mathematical model in which biological elements, such as molecules, are represented as nodes (also known as vertices), and defined relationships between these elements are represented as links (or edges) (de Anda-Jáuregui et al., 2015). As a mathematical object, networks (also known as graphs) can be analyzed using well-established algorithms (Albert and Barabási, 2002; Barabási et al., 2011); in this sense, they offer a general framework for the study of natural phenomena. A biological network will have, based on the existing relationships between the elements that compose them, structural and topological characteristics that reflect underlying biological properties (Barabasi et al., 2004).

Network pharmacology expands on the network biology paradigm to address the problem of identifying pairs of drugs and targets that are clinically successful, maximizing therapeutic effects and minimizing toxicity (Harrold et al., 2013). It provides a framework which may be used to overcome limitations of other methods for drug exploration such as those based on phenotypic effects or those based only on chemical structure; it serves as a tool to integrate knowledge from the pharmacological and genomic spaces (Zhao and Li, 2010). Network pharmacology is becoming more relevant as the traditional single target pharmacological model shifts toward a model that (1) considers the perturbation of multiple biological entities in a disease (Kibble et al., 2016) and (2) considers functional targets as more suitable than molecular targets for effective drug therapies (Hopkins, 2008).

Pharmacological phenomena fundamentally involve interactions between elements of different origin and nature; for example, interactions between a drug and its biological targets or observable biological effects, either therapeutic or toxic. With this in mind, a suitable network model to study the pharmacological space can be a *bipartite network*, in which nodes represent elements of two distinct classes and relationships exist only between elements of different classes (Guillaume and Latapy, 2006).

In this work, we modeled the relationships among anti-inflammatory drugs, their effects at the gene perturbation and pathway perturbation level, and their associated adverse reactions from a network perspective. Based on the topological properties derived from these networks, we propose strategies to prioritize anti-inflammatory drug repurposing, quantify potential side effect risk, and identify possible pathway perturbation-related mechanisms associated with side effects in the context of specific anti-inflammatory drug sets This provides a toolset to identify anti-inflammatory drugs that are candidates with better repurposing opportunities.

## Materials and methods

**Figure 1:**
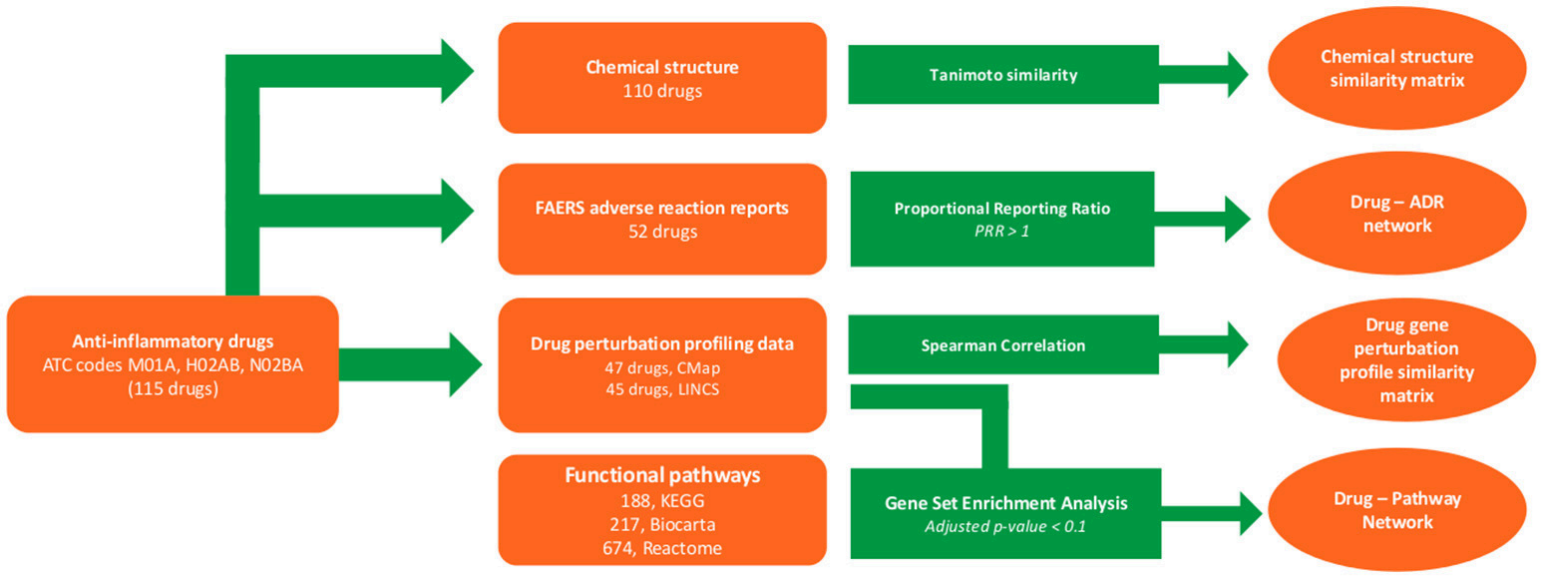
Methodological pipeline. The workflow used here consists of the following steps. First, drugs with anti-inflammatory therapeutic indications were identified. Their chemical structures, drug perturbation profiles, and adverse drug reactions (ADRs) were then collected from databases, along with functional pathway information. These collected data were used to construct similarity matrices (based on chemical or gene perturbation), drug-pathway perturbation networks, and drug-ADR networks.

In this work, anti-inflammatory drugs were identified, and chemical structures, gene perturbation profiles, and ADR report data were collected. Using these data, similarity matrices and bipartite networks were generated. Figure 1 illustrates our overall workflow.

### Data

#### List of anti-inflammatory drugs

Anti-inflammatory drugs were identified using the anatomical therapeutic chemical (ATC) classification system. Drugs with ATC codes M01A (anti-inflammatory and antirheumatic products, non-steroids), H02AB (corticosteroids for systemic use, plain), and N02BA (salicylic acid and derivatives) were selected. Drug names were normalized to the official DrugBank name or generic names to be consistent and comparable across various datasets in the current study. Our in-house drug-name normalization pipeline was used as previously described (Hur et al., 2014). The chemical structures of the anti-inflammatory drugs of interest were retrieved from PubChem (Kim et al., 2016) and DrugBank (Wishart et al., 2008) databases in simplified molecular-input line-entry system (SMILES) format.

#### Drug-gene perturbation profiles

Two large-scale drug-perturbation gene expression datasets containing genome-wide transcriptional expression data from cultured human cell lines treated with bioactive small-molecules were included. The first dataset is the Connectivity Map (CMap, version 02), with over 7,000 expression profiles representing 1,309 compounds in five cultured human cell lines that were measured using Affymetrix human genome U133A (HGU133A) arrays. The second is the L1000 dataset from the Library of Integrated Network-based Cellular Signatures (LINCS) project (Duan et al., 2014), which measured gene expression changes after treatment of 83 human cells with over 20,000 small-molecule compounds using the L1000 platform (https://www.ncbi.nlm.nih.gov/geo/query/acc.cgi?acc=GPL20573). The LINCS L1000 dataset is composed of two publicly available releases with Gene Expression Omnibus (GEO; https://www.ncbi.nlm.nih.gov/geo/) accession numbers GSE92742 (LINCS phase I) and GSE70138 (LINCS phase II), which were merged into a single LINCS dataset in our study.

Probe data were aggregated to the gene level when applicable (HGU133A platform). In cases of more than one experimental condition involving a drug of interest, we merged perturbation profiles using the Kruskal–Borda merging algorithm (Iorio et al., 2010) to generate a consensus profile. Considering the use of different quantification platforms for both datasets, analyses using these were conducted independently for the first CMap dataset (hereafter referred to as CMap) and the dataset belonging to the LINCS project (hereafter referred to as LINCS).

#### Drug adverse reaction information

Pharmacovigilance information from the Food and Drug Administration (FDA) adverse event reporting system (FAERS) (FDA, 2014) from 2012 to the third quarter of 2016 was used. The FAERS data were downloaded and processed using the FAERS package (https://github.com/mlbernauer/FAERS). Drug names were normalized to generic names using the FAERS package as well as our name-normalization pipeline. Drugs with multiple single active ingredients were excluded from further analyses. ADRs were normalized to the MedDRA release 20.0 preferred terms (PTs) and mapped to MedDRA higher level terms (HLT) (https://www.meddra.org/).

#### Pathway collection

Kyoto Encyclopedia of Genes and Genomes (KEGG) pathways (Kanehisa et al., 2014), as well as Reactome (Fabregat et al., 2016) and BioCarta (Nishimura, 2001) databases retrieved from the Broad Institute's Molecular Signature database (Subramanian et al., 2005) category C3 were used.

### Similarity matrices construction

#### Drug-drug structural similarity

Drug structures in SMILES format were used to obtain a unique atom pair library (Chen and Reynolds, 2002) for each anti-inflammatory drug. For each pair of anti-inflammatory drugs, Tanimoto similarity was calculated using the ChemmineR package in R (Cao et al., 2008).

#### Drug-drug gene perturbation profile similarity

For each collection of drug perturbation profiles (CMap and LINCS), similarity in gene-level effects between drugs was analyzed. To do so, Spearman similarity between the ranked gene perturbation profiles was calculated for each pair of drugs. The CMap set and the LINCS set were analyzed separately.

#### Non-supervised hierarchical clustering

Drugs were clustered hierarchically in both the structural and perturbation profile similarity matrices. Briefly, a set of dissimilarities were generated from the matrices, and each element (drug) was assigned to a cluster; iteratively, each pair of most similar clusters was merged, until a single cluster was obtained. Clustering was performed using the complete linkage method with the *hclust* function in the R *stats* package (version 3.4.2).

### Bipartite network construction

#### Drug-pathway perturbation network

Any drug may potentially have effects beyond their canonical targets. Some of these effects manifest in the perturbation of system-scale biological pathways, which are evident as a significantly coordinated change in the genetic expression of the molecules involved in it. To identify possible system-wide effects of anti-inflammatory drugs, bipartite networks of drugs and pathway perturbations were constructed. For each gene perturbation profile associated with a drug in our datasets, we used a gene set enrichment analysis (GSEA) (Subramanian et al., 2005) to identify the pathways exhibiting significant alterations attributable to anti-inflammatory drug treatment. The fast GSEA (FGSEA) (Sergushichev, 2016) implementation was used with 50,000 permutations per analysis, with a significance cut-off set as an adjusted *p* < 0.1.

Results of the enrichment analysis were integrated into a bipartite network composed of anti-inflammatory drugs and significantly enriched pathways in at least one drug. An edge was established between a drug and a pathway if the drug treatment resulted in significant enrichment of the pathway (adjusted *p* < 0.1). The edges in this network were unweighted, as any pathway with an adjusted *p* value below the threshold was determined to be significantly enriched.

#### Drug-ADR network

ADRs were linked to those drugs most likely to produce them. The proportional reporting ratio (PRR) (Evans et al., 2001) is a statistical measure of relative risk for a given ADR extracted from pharmacovigilance data that compare the specific frequency of an ADR under a given condition (i.e., drug treatment) against the overall frequency of the ADR. PRRs for ADRs between anti-inflammatory drugs and non-anti-inflammatory drugs were first calculated. ADRs with PRRs > 1 were then selected: these included ADRs more likely to be associated with anti-inflammatory drugs than with other drugs. ADRs more likely to be associated with specific anti-inflammatory drugs were identified by calculating the PRR for the previously selected ADRs among anti-inflammatory drugs only. Calculations were done using the R package PhViD (Ahmed et al., 2010).

The FAERS dataset presents ADRs in terms of MedDRA Preferred Terms. Considering that there are 22,500 different PTs, these were mapped to their corresponding MedDRA HLT. A bipartite network was constructed in which the first set of nodes are drugs and the second set of nodes are ADRs represented as HLTs. An edge was established between a drug and an HLT if there was at least one PT associated with the HLT and a PRR > 1. Since PRR is a measure of relative risk, the edges in this network were considered to have a weight; the weight of an edge is the sum of the PRRs between the drug and the set of PTs that can be associated with a particular HLT.

#### Pathway-ADR network

Through the merging and projection of the drug-pathway network and the drug-ADR network, a pathway-ADR network was generated. In this network, a pathway was connected to an ADR if there was at least one drug connected to both. Edges in the network have a weight, which represents the number of drugs through which a pathway and an ADR are connected.

#### Network analysis

For each network, basic network properties such as number nodes, number of edges, and network density were calculated. Centrality measures such as degree, clustering coefficient, and redundancy coefficient (Latapy et al., 2006) were calculated for each set of nodes using the R package Igraph (Csardi and Nepusz, 2006) and the Python package NetworkX (Hagberg et al., 2008). Supplementary File 1 contains GML files for each network.

## Results

### Drug-drug structural similarity

**Figure 2:**
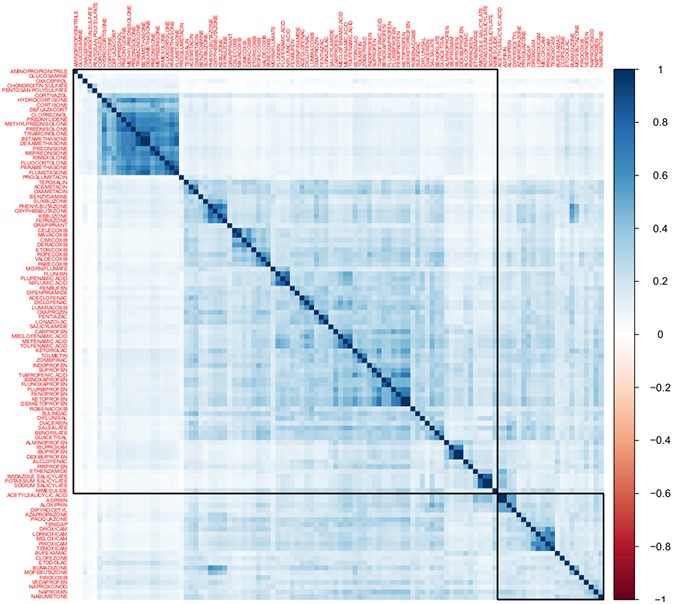
Heatmap of Tanimoto structural similarity. This heatmap represents structural similarities between 110 anti-inflammatory drugs. The color intensity is proportional to the similarity between the chemical structure of two drugs measured using the Tanimoto coefficient. Drugs were ordered using non-supervised hierarchical clustering. Clusters in this matrix contain drugs with close chemical structures. For instance, a large cluster (purple outline) contains hydrocortisone and other drugs derived from it.

A total of 114 anti-inflammatory drugs were identified based on our ATC inclusion criteria as of July 2017. We obtained drug structure data for 110 of these drugs from DrugBank and PubChem. Figure 2 shows a heatmap representing the structural similarities among these compounds. Supplementary File 2 contains the corresponding data matrix. In this heatmap, drugs are arranged through non-supervised hierarchical clustering. These clusters are composed of drugs with similar structures, such as drugs that are derived from a lead molecule. For instance, there is a large cluster which contains steroidal anti-inflammatory drugs that are similar in structure to hydrocortisone; this cluster also has the highest similarity values. Non-steroidal anti-inflammatory drugs are grouped in smaller clusters, consistent with the more diverse structures found in this group.

### Gene perturbation similarity of anti-inflammatory drugs

**Figure 3:**
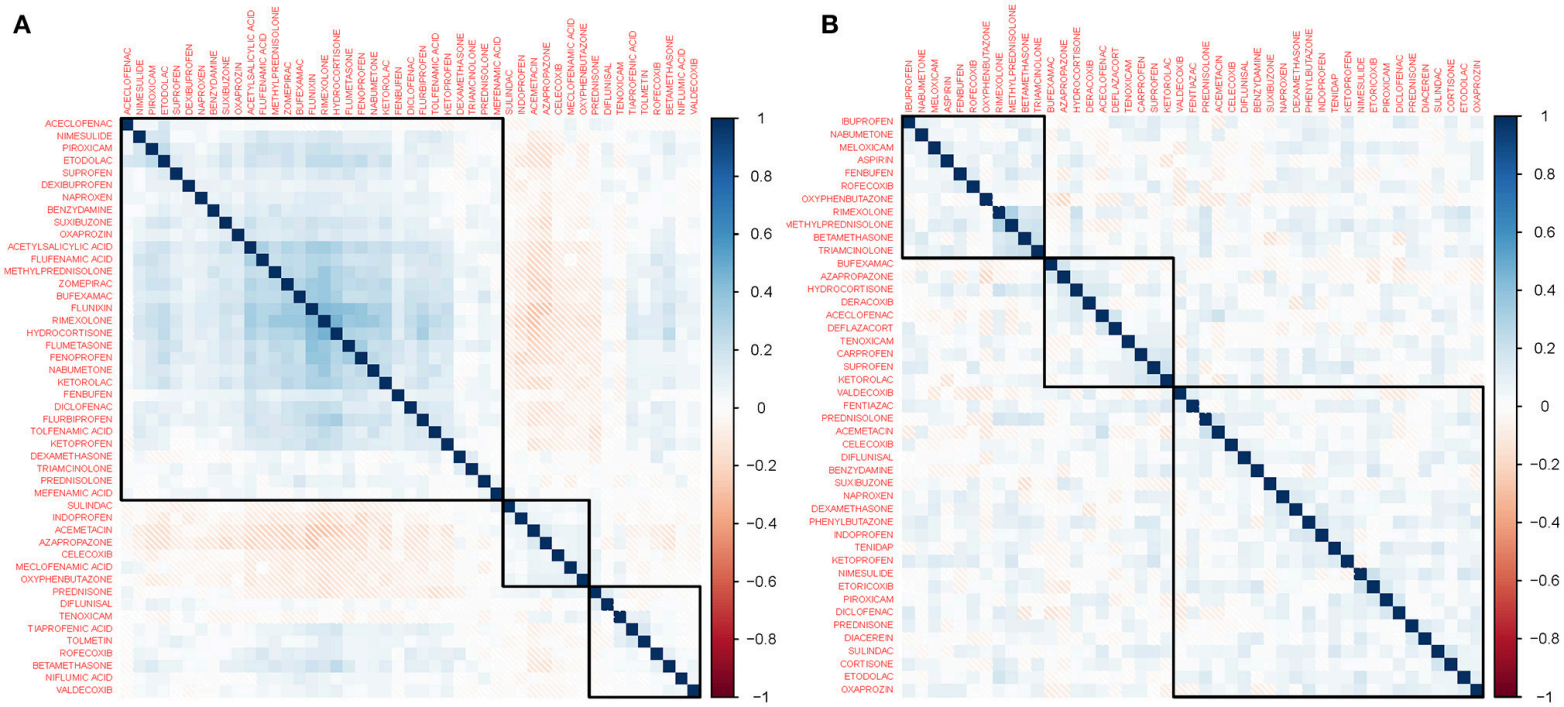
Heatmap of gene perturbation similarity. These heatmaps represent similarities in gene expression profiles induced by drugs obtained from the **(A)** Connectivity Map (CMap) and **(B)** Library of Integrated Network-based Cellular Signatures (LINCS) datasets. The color intensity is proportional to the similarity between the gene expression profiles of two drugs measured using the Spearman correlation. Drugs are ordered using non-supervised hierarchical clustering. In both panels, a larger cluster containing most drugs and two smaller clusters are shown. The clusters comprise drugs that do not necessarily share structural similarities.

Approximately 40% of the 114 anti-inflammatory drugs were included in the CMap and LINCS datasets (47 and 45 drugs, respectively). Only 25 drugs were found in both datasets. The heatmaps in Figure 3 illustrate the similarities between drugs in terms of gene perturbation based on the Spearman correlation of their ranked gene perturbation profiles; color intensity is proportional to the correlation value. Supplementary File 1 contains the corresponding data matrix.

In the CMap dataset, a group of 31 drugs formed a large cluster based on their gene perturbation effects. The rest of the drugs are in smaller clusters of 9 and 7 drugs. These drugs belong to both steroidal and non-steroidal classes of anti-inflammatory drugs, showing a similarity in perturbation effects not limited by structural features. Although we identified similar clusters using the LINCS dataset, the overall similarities between drug profiles were lower, as visualized in the heatmap via less color intensity.

### Drug-pathway perturbation network

Networks linking drugs to functional pathways were constructed based on their effects at the gene perturbation level. These networks allow the identification of biological functions that can be affected by a given drug and the identification of drugs that can affect a given biological function. The GSEA algorithm was used to identify which functional pathways are affected by each anti-inflammatory drug. Supplementary Figure 1 shows the resulting bipartite networks using a hive plot visualization. In this visualization, nodes are arranged along axes and edges and are represented by Bezier curves. Supplementary Figure 1A shows the CMap-derived drug-pathway perturbation network, which is dominated by the largest connected component (a subgraph composed of a set of nodes in which any pair of nodes is connected by a path, and there is no path connecting them to a node outside the subgraph), represented by a drug-containing axis and a pathway-containing axis densely populated with edges between them; this component contains most pathways perturbed by anti-inflammatory drugs. A second component containing pathways and two drugs is also shown. Twenty drugs with no pathway effects were found and are arranged along an axis with no edges. Supplementary Figure 1B shows the LINCS-derived drug-pathway perturbation network. Again, this network was dominated by the largest connected component concentrating most of the perturbed pathways, along with four smaller pathway-containing components and 7 disconnected drugs. The properties of these networks are summarized in Table 1.

**Table 1:** Graph parameters for drug-pathway perturbation networks.

	**CMap**	**LINCS**
Nodes, drugs	47	45
Nodes, pathways	304	302
Edges	760	624
Network density	0.05	0.05
Average clustering coefficient, drugs	0.14	0.08
Average clustering coefficient, pathways	0.39	0.44

In both networks, most drugs with at least one pathway target belong to the same largest connected component. The most noticeable exceptions are azapropazone and acemetacin in the CMap-based network. These two drugs form a lone connected component where they both target 11 pathways related to signaling [including g protein-coupled receptor (GPCR) and calcium signaling] as well as xenobiotic metabolism, setting them apart from the rest of the anti-inflammatory drug space in terms of system-wide effects. In the case of the LINCS-based network, there were three small components comprising single drug-pathway pairs [celecoxib and the trefoil factor (TFF) pathway; bufexamac and valine, leucine, and isoleucine degradation; and nabumetone and WNT signaling], as well as a component formed by a drug and two pathways [valdecoxib and the extracellular signal-regulated kinase (ERK) pathway and acyl chain remodeling of phosphatidylglycerol].

**Figure 4:**
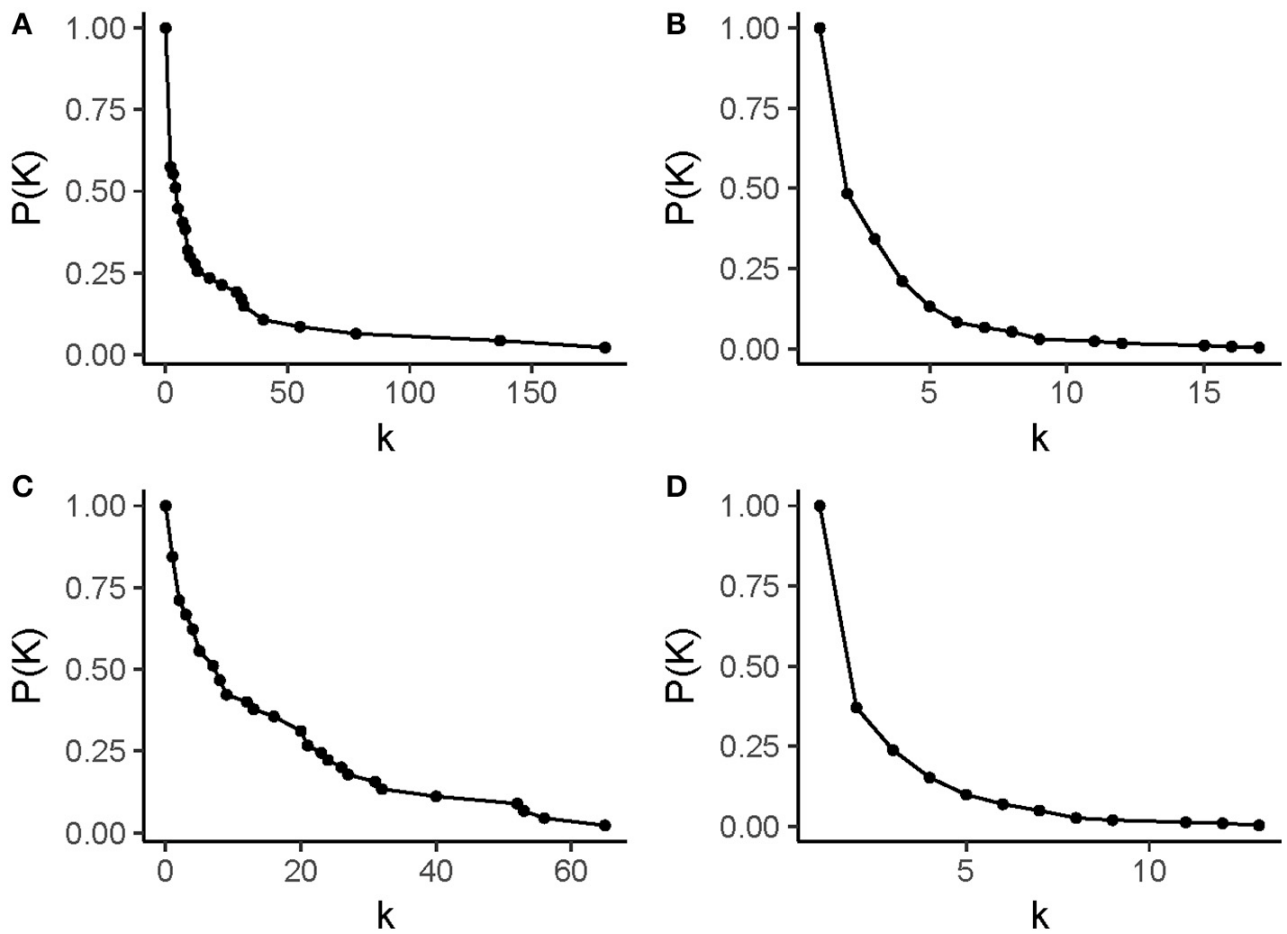
Degree distributions in drug-pathway networks. **(A)** drug nodes in the CMap-based network, **(B)** pathway nodes in the CMap-based network, **(C)** drug nodes in the LINCS-based network, and **(D)** pathway nodes in the LINCS-based network. In each panel, the x-axis represents a degree value (*k*) and the y-axis represents the complementary cumulative frequency for a value of *k, P*(*K*). Drug nodes have degree values that range from 0 to 180 in the CMap-based network and 0 to 65 in the LINCS-based network. Pathways have degree values that range from 1 to 17 in the CMap-based network and 1–13 in the LINCS-based network. It should be noted that in all degree distributions, nodes with higher degree values are fewer than nodes with low degree values.

Figure 4 shows the degree distribution in these networks. The degree of a node refers to its number of adjacent edges; it is one of the defining measures of any network. The degree value in these drug-pathway perturbation networks has different meanings for each type of node. A “drug degree” represents the capacity of a given drug to have multiple functional effects at the pathway level. Meanwhile, a “pathways degree” represents the susceptibility of a given pathway to be targeted by anti-inflammatory drugs. It should be noted that the drug degree distribution and the pathway degree distribution are different in each network: first, the drug degree distribution has a larger range than the pathway degree distribution; second, the frequency of drug nodes with a single neighbor is higher than that of pathway nodes with a single neighbor. These plots also show that these networks are predominantly populated with pathways and drugs that have few neighbors, whereas highly connected nodes are scarce.

Pathway degrees range from 1 to 13 in the LINCS network and 17 in the CMap network; there are no pathways with a degree of 0, as expected given the network construction model. Interestingly, the most connected pathway was the “cell cycle” pathway from the Reactome database in both networks, making it the most susceptible pathway to anti-inflammatory drugs. However, there was not a single pathway that was perturbed at the gene expression level by all the anti-inflammatory drugs. Drug degrees range from 0 to 65 in the LINCS-based network and from 0 to 180 in the CMap-based network; drugs with the highest degrees were azapropazone and rimexolone, respectively. It must be noted that in the case of drug nodes, the network construction model allows for the presence of nodes with a degree of 0.

Supplementary Figure 2 shows clustering coefficient distributions for each network. The clustering coefficient of a node in a bipartite network is a measure of how likely it is, on average, for a given node to share neighbors with others (Latapy et al., 2006). In the case of these networks, a higher clustering coefficient for a drug node can be interpreted as the likelihood that pathways affected by a given drug are affected by another drug. In contrast, for pathway nodes, a higher clustering coefficient represents the likelihood of finding another pathway that is susceptible to a similar set of drugs. The clustering coefficient for drugs in both networks is below 0.2 for all drugs in the largest connected component. A related concept is node redundancy. This parameter measures whether the removal of a given node from the bipartite graph leads to the disconnection of two of their neighbors. Drug node redundancy can represent the *uniqueness* of pathway effects, with highly non-redundant drugs being those that are able to affect pathways untargeted by other anti-inflammatory drugs. The distribution of redundancy values is shown in Supplementary Figure 3.

### Drug-ADR network

Relationships between anti-inflammatory drugs and ADRs contained in the FAERS dataset were used to generate a bipartite network. This network allows the identification of possible adverse effects for a given drug and drugs that are associated to a given adverse effect. We first identified those side effects more likely to be associated with anti-inflammatory drugs; individual drugs more likely to be associated with particular side effects were then identified. The resulting bipartite network is presented in Supplementary Figure 4 as a hive plot comprising a single large component containing all anti-inflammatory drugs studied and their side effects. The transparency of the Bezier lines is proportional to the weight of the edge, representing the ADR risk for the associated drug. The statistics describing this network are found in Table 2.

**Table 2:** Graph parameters for the drug-adverse drug reaction (ADR) network.

	**Drug—ADR**
Nodes, drugs	52
Nodes, ADRs	1,175
Edges	9,597
Network density	0.16
Average clustering coefficient, drugs	0.13
Average clustering coefficient, ADRs	0.17

**Figure 5:**
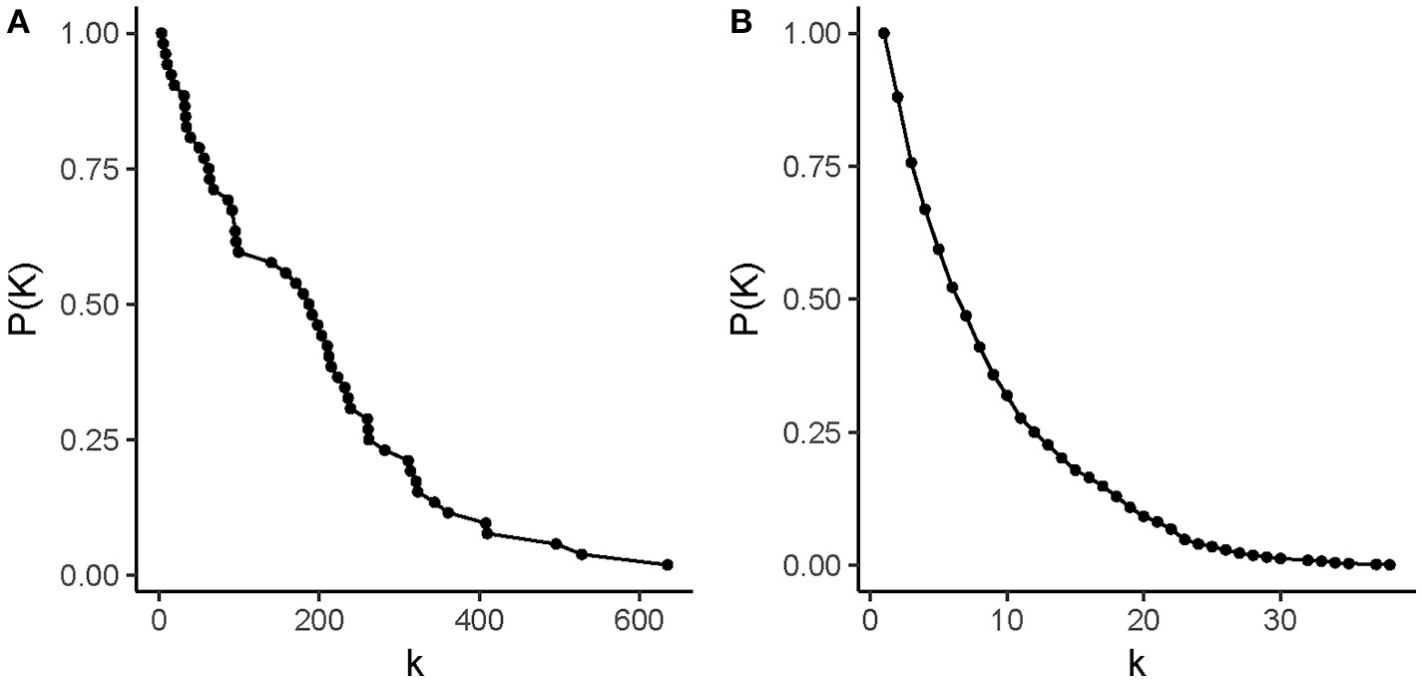
Degree distributions in drug-adverse drug reaction (ADR) networks. This figure illustrates the degree distribution of **(A)** drug nodes and **(B)** ADR nodes. In each panel, the normalized, complementary cumulative degree frequency distribution is presented. Drug nodes have degree values that range from 0 to 635, while pathways have degree values that range from 1 to 38.

Figure 5 shows the cumulative frequency distribution for degree. The ADR degrees ranged from 1 to 38; the highest degree was associated with a non-specific “general signs and symptoms, not elsewhere classified.” Further, the drug degrees ranged from 3 for flufenamic acid to 635 in the case of prednisolone, suggesting that all anti-inflammatory drugs are more frequently associated with side effects than other drugs. Supplementary Figures 5, 6 show the previously discussed parameters of clustering coefficient and redundancy. In the case of drugs, the clustering coefficient increased up to a value of 0.18, while for ADR nodes, the maximum clustering coefficient was 0.23.

Another centrality measure only defined in weighted networks is node strength. The strength of a node in a weighted network is defined as the sum of weights for all adjacent edges (Barrat et al., 2004). In this network, weights represent the risk of a given ADR for a drug. Node strength for each drug can be used as a general measure of relative risk for any side effect, regardless of severity, to manifest in a treatment with a given drug. The strength distribution for drug nodes is shown in Supplementary Figure 7.

### Pathway-ADR network

This network represents links between functional pathways and ADRs in the anti-inflammatory setting resulting from a projection of the drug-pathway and drug-ADR networks. Here, functional pathways and ADRs are linked if both are associated with at least one shared anti-inflammatory drug; the edge weight in these networks is the number of shared drugs between a pathway and an ADR. Table 3 contains descriptors related to these networks.

**Table 3:** Graph parameters for pathway-adverse drug reaction (ADR) networks.

	**CMap**	**LINCS**
Nodes, ADRs	1,160	1,165
Nodes, pathways	304	302
Edges	48,430	95,311
Network density	0.137	0.27
Average clustering coefficient, ADRs	0.27	0.28
Average clustering coefficient, pathways	0.26	0.25

These networks help identify possible associations between functional pathways and ADRs in the context of anti-inflammatory drugs that can be used to generate hypotheses regarding the underlying mechanisms of the side effects associated with these drugs. For instance, “*eye and eyelid infection”* was a side effect identified with a high number of connections to three Reactome pathways, including cell cycle, cell cycle mitotic, and DNA replication using the CMap-derived pathway-ADR network. There were four drugs through which these ADR and pathways are connected: hydrocortisone, ketoprofen, methylprednisolone, and rimexolone. Each of these drugs can be found in ophthalmic medications. The connection between these drugs and this ADR can therefore be explained by the therapeutic indication of these drugs. Meanwhile, the mechanism through which these drugs may affect these pathways is not known, although it is important to notice that cell cycle pathways are affected by several anti-inflammatory drugs, as mentioned previously in section Drug-Pathway Perturbation Network. Without further experimental confirmation, further discussion on the role of these pathways regarding the ADR would be speculative. However, a putative role of the perturbation of these pathways in the ophthalmic infection condition, at least in the context of treatment with this set of drugs, is an example of a non-trivial insight that can be generated through a network-based analysis and which may drive novel experimental research.

## Discussion

In this work, we explored relationships among anti-inflammatory drugs based on parameters such as chemical structure similarity, gene perturbation, functional pathway perturbation, and ADRs. Network models were constructed and examined based on these features, which can provide further insights into the relationship of these drugs with their pharmacological effects; we found that functional features, such as perturbed pathways and ADRs, are more informative for this purpose. Through the exploration of drug centrality and functional features in bipartite networks, we identified drugs that could be prioritized for potential repurposing. Finally, we found associations between drug effects at the pathway level and side effects, which may point to underlying mechanisms for these effects in the anti-inflammatory setting.

Structural and perturbation similarities are good comparison parameters for the general drug space (Iorio et al., 2010; Lo et al., 2015). As the anti-inflammatory drug space is already a subset of the greater drug space that shares common therapeutic applications, differences (structural and at the raw gene perturbation level) are probably much less likely to be observed in the anti-inflammatory drug space than in the general drug space. It is difficult to determine a non-arbitrary cut-off value to generate an insightful network model based on similarities in the structural or gene perturbation profile. However, the bipartite models proposed in this work do not have this limitation, leading to a more descriptive landscape of the functional effects of these therapeutic drugs. Around 300 functional pathways that are potentially susceptible to modulation through anti-inflammatory drug use were identified, along with nearly 1,200 ADRs. Therefore, this network-based approach is presented as more suitable for the exploration of a limited section of the pharmacological space than studies based on chemical structural features alone.

The structure of these networks provides insight regarding the similarities and differences between anti-inflammatory drugs. Drug-pathway networks are dominated by the largest connected component in each network. Drugs that are part of the smaller components affect pathways that are not susceptible to the effects of other drugs. Drugs that have common effects that are shared with many other drugs in the network have higher clustering coefficients and redundancy values. Drugs whose pathway effects are completely redundant such that there is another drug with the exact same set of pathway effects are rare, which is consistent with the coexistence of these drugs in current medical practice. The drug-ADR network exclusively comprises a single connected component, indicating a widespread overlap in potential side-effects between these drugs.

The connectivity of a drug in the networks presented in this manuscript is indicative of the drug's effects, either therapeutic, in the context of the pathway networks, or toxic, in the case of the ADR network. In the case of drug-pathway networks, node degree is a measure of connectivity; high-degree drugs in these networks affect more functional pathways. Some drugs were found to have no significant effects on pathways, appearing as drugs with a degree of 0. Evidently, this does not mean that these drugs have no pharmacological effects; rather, it is indicative of no observable activity at the gene expression level (as tested in cell cultures), making them comparatively less likely to exhibit other system-wide effects than other more connected anti-inflammatory drugs. In the context of ADRs, there are two complementary connectivity measures for each drug: degree indicates the number of possible side effects of a given drug, while node strength is a measure of relative risk for any side effect. The use of network-based metrics allows for a simple and generalizable categorization of these drugs based on their possible biological effects.

**Figure 6:**
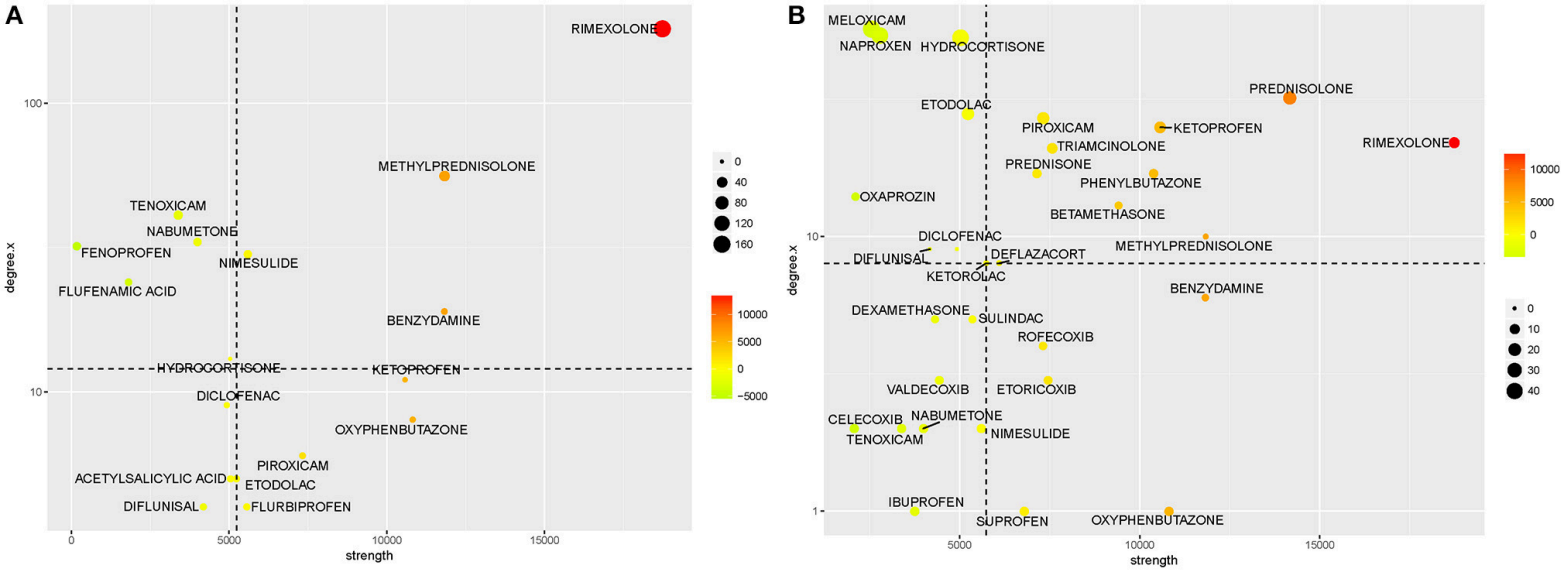
Toxic and therapeutic potential of the anti-inflammatory drug space. In these figures, drugs are classified based on their risk of producing an adverse drug reaction (ADR, measured as the node strength from the drug-ADR network) and their potential alternative therapeutic uses [measured as the degree in a drug-pathway network, either derived from **(A)** the Connectivity Map (CMap) or **(B)** Library of Integrated Network-based Cellular Signatures (LINCS)]. Each axis is divided by its median value. Point color and size are proportional to position in the x and y axes. Following this classification, drugs in the upper, left quarter are the best repositioning candidates, as they have the most effects on alternative pathways and the least risk for ADRs.

Drug connectivity in the networks presented in this manuscript can be useful in the context of drug repurposing. It is possible to use the degree of a drug node in the drug-pathway network as a measure of the number of potential alternative therapeutic targets. The node strength of a drug in the drug-ADR network is an indicator of its general risk for generating a side effect. Using these two axes, the anti-inflammatory space can be divided into four groups as shown in Figure 6. The drug space with the highest pathway targets and the lowest side effect strengths would be the best suited to identify drug candidates for repurposing. For illustration purposes, each axis is divided by its median value; drugs with higher than median pathway effects and lower than median side effect strength appear in the upper, left quadrant. Arguably, these drugs are more likely to be successfully repurposed, and there are literature reports that support this hypothesis. For instance, the use of naproxen as a cytotoxic drug in urinary bladder cancer has been reported (Kim et al., 2014). Other repurposed applications have been proposed for meloxicam in the treatment of non-Hodgkin's lymphoma (Nugent et al., 2016; Chartier et al., 2017), etodolac for the treatment of breast cancer (Yang et al., 2014), tenoxicam for the treatment of tuberculosis (Maitra et al., 2016), hydrocortisone in the treatment of Alzheimer's Disease (Zhang et al., 2015), flufenamic acid for the treatment of *Salmonella* infection (Ekins et al., 2011; Preethi et al., 2016), fenoprofen as a melanocortin receptor allosteric enhancer (Montero-Melendez et al., 2017), and nabumetone (Shameer et al., 2017).

The approach described in this work is currently limited by constraints related to the availability of drug effect information. The CMap and LINCS drug perturbation datasets, which are some of the most extensive and have robust and reproducible generation methodologies, were used. Nonetheless, the coverage of tested drugs is far from complete, which is reflected in the composition of the resulting networks. In the case of pharmacovigilance information, it is important to consider the inherent limitations associated with drug monitoring, as well as limitations in the reporting infrastructure and changes in prescription habits, which may affect the availability of adverse effect information for drugs. As such, it is important to note that the approach presented here is implemented in an exploratory setting. The aforementioned limitations may lead to the identification of false positive leads, which can only be overcome with further, well-defined experimental research.

Since our focus in this work was a well-defined subset of the therapeutic drug space, we suggest that the relationships identified to the functional features identified here may be specific only to this subset of drugs. We do not know whether the topologies of similarly constructed bipartite networks for other drug classes, or for the larger drug space, will share similarities, as this was beyond the scope of the present work.

## Conclusions

In this work, we explored a subset of the pharmacological space integrated by anti-inflammatory drugs. We identified relationships between these drugs based on their chemical structure and effects on gene expression, as well as physiological effects such as alteration of functional pathways and the onset of adverse reactions. We integrated these into bipartite network models that we analyzed to identify topological properties related to these relationships. We showed that using the bipartite network model provides advantages for the exploration of the anti-inflammatory drug space that are not possible by using other analysis strategies. We expect to expand our model as new high-throughput drug screening protocols generate further information regarding drug effects.

We suggest that the present work provides a framework to explore functional effects of certain therapeutic classes. We focused on the anti-inflammatory space, considering its notable clinical importance. We demonstrated that it is possible to gain insights relevant to pharmaceutical research using these models, which can be integrated to drug repurposing and drug combination pipelines, as well as to the clinical setting. This will provide further criteria for the selection of optimal anti-inflammatory therapies. Finally, we exemplified an application integrating different sources of pharmacological information into network models for drug repurposing.

## Author contributions

GdA-J: developed and implemented the network models and analyses; KG: implemented pipelines for database management and visualization; GdA-J, BM, KG, and JH: contributed to the analysis and discussion of results. All authors approve of the final manuscript.

### Conflict of interest statement

The authors declare that the research was conducted in the absence of any commercial or financial relationships that could be construed as a potential conflict of interest.

## Supplementary material

Supplementary File 1 A compressed file containing all networks as graph markup language (GML) files.

Supplementary File 2 A compressed file containing all similarity matrices as comma-delimited files.

Supplementary Figure 1 Graph visualization of the drug-pathway perturbation networks. In these visualizations, networks are presented as hive plots. Nodes representing drugs and pathways are arranged along axes, and edges representing perturbation of a pathway by a drug are shown as Bezier curves. **(A)** shows a network derived from CMap data containing two connected components; the largest one (comprising the orange drug axis and the yellow pathway axis) contains the majority of pathway perturbing drugs and perturbed pathways. A second component contains two drugs (pink axis) perturbing 11 pathways. Drugs for which no pathway effects were found are arranged along the green axis. **(B)** shows a network derived from LINCS data that is dominated by a large connected component with 4 smaller components containing drugs and perturbed pathways. Drugs for which no pathway effects were found are arranged along the purple axis.

Supplementary Figure 2 Clustering coefficient in drug-pathway networks. **(A)** Drug nodes in the CMap-based network, **(B)** pathway nodes in the CMap-based network, **(C)** drug nodes in the LINCS-based network, and **(D)** pathway nodes in the LINCS-based network. In each panel, the normalized, cumulative clustering coefficient frequency distribution is presented. Drug nodes have degree values that range from 0 to 0.45 in the CMap-based network and 0 to 0.19 in the LINCS-based network. Pathways have degree values that range from 0 to 0.75 in the CMap-based network and 0 to 1 in the LINCS-based network.

Supplementary Figure 3 Redundancy coefficient in drug-pathway networks. **(A)** Drug nodes in the CMap-based network, **(B)** pathway nodes in the CMap-based network, **(C)** drug nodes in the LINCS-based network, and **(D)** pathway nodes in the LINCS-based network. In each panel, the normalized, cumulative redundancy frequency distribution is presented.

Supplementary Figure 4 Graph visualization of the drug-adverse drug reaction (ADR) network. A hive plot representation of the drug-ADR network is shown. This network comprises 52 drug nodes and 1,227 ADR nodes, with 9,597 links between them. Drugs nodes are arranged along the blue axis, while ADR nodes are arranged along the red axis. Edges between them are shown as Bezier curves. The transparency of each line is proportional to the edge strength. The network comprises a single large connected component containing all drugs and pathways.

Supplementary Figure 5 Clustering coefficient in drug-adverse drug reaction (ADR) networks. This figure illustrates the clustering coefficient distribution of **(A)** drug nodes and **(B)** ADR nodes. In each panel, the normalized, cumulative clustering coefficient frequency distribution is presented. Drug nodes have a maximum value of 0.18, while pathway nodes have a maximum value of 0.24.

Supplementary Figure 6 Redundancy coefficient in drug-adverse drug reaction (ADR) networks. This figure illustrates the redundancy coefficient distribution of drug nodes **(A)** drug nodes and **(B)** ADR nodes. In each panel, the normalized, cumulative redundancy frequency distribution is presented for drug and ADR nodes, respectively.

Supplementary Figure 7 Node strength in drug-adverse drug reaction (ADR) networks for drug nodes. The normalized, cumulative strength distribution for drug nodes is shown, with a range of 0 to 85,591.61. A node's strength in a weighted network is the sum of the weights of all adjacent edges. In this network, an edge weight represents the risk of an ADR for a given drug. Therefore, a drug's node strength is a measure of general risk for a drug.
