# Supplementary figures and images for: Exploration of the Anti-Inflammatory Drug Space Through Network Pharmacology: Applications for Drug Repurposing
Source: Front Physiol. 2018 Mar 1;9:151. doi: 10.3389/fphys.2018.00151 (PMC5838628; doi:10.3389/fphys.2018.00151)

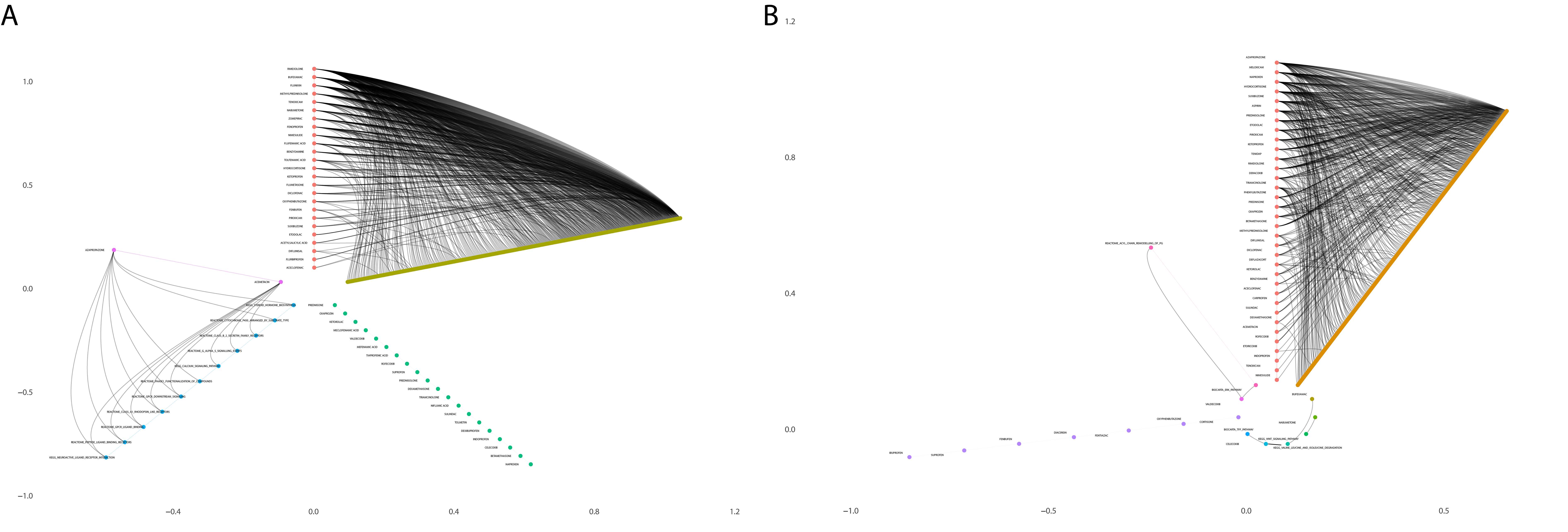

Supplement: Supplementary Figure 1 — Graph visualization of the drug-pathway perturbation networks. In these visualizations, networks are presented as hive plots. Nodes representing drugs and pathways are arranged along axes, and edges representing perturbation of a pathway by a drug are shown as Bezier curves. (A) shows a network derived from CMap data containing two connected components; the largest one (comprising the orange drug axis and the yellow pathway axis) contains the majority of pathway perturbing drugs and perturbed pathways. A second component contains two drugs (pink axis) perturbing 11 pathways. Drugs for which no pathway effects were found are arranged along the green axis. (B) shows a network derived from LINCS data that is dominated by a large connected component with 4 smaller components containing drugs and perturbed pathways. Drugs for which no pathway effects were found are arranged along the purple axis. [file Image1.TIF]

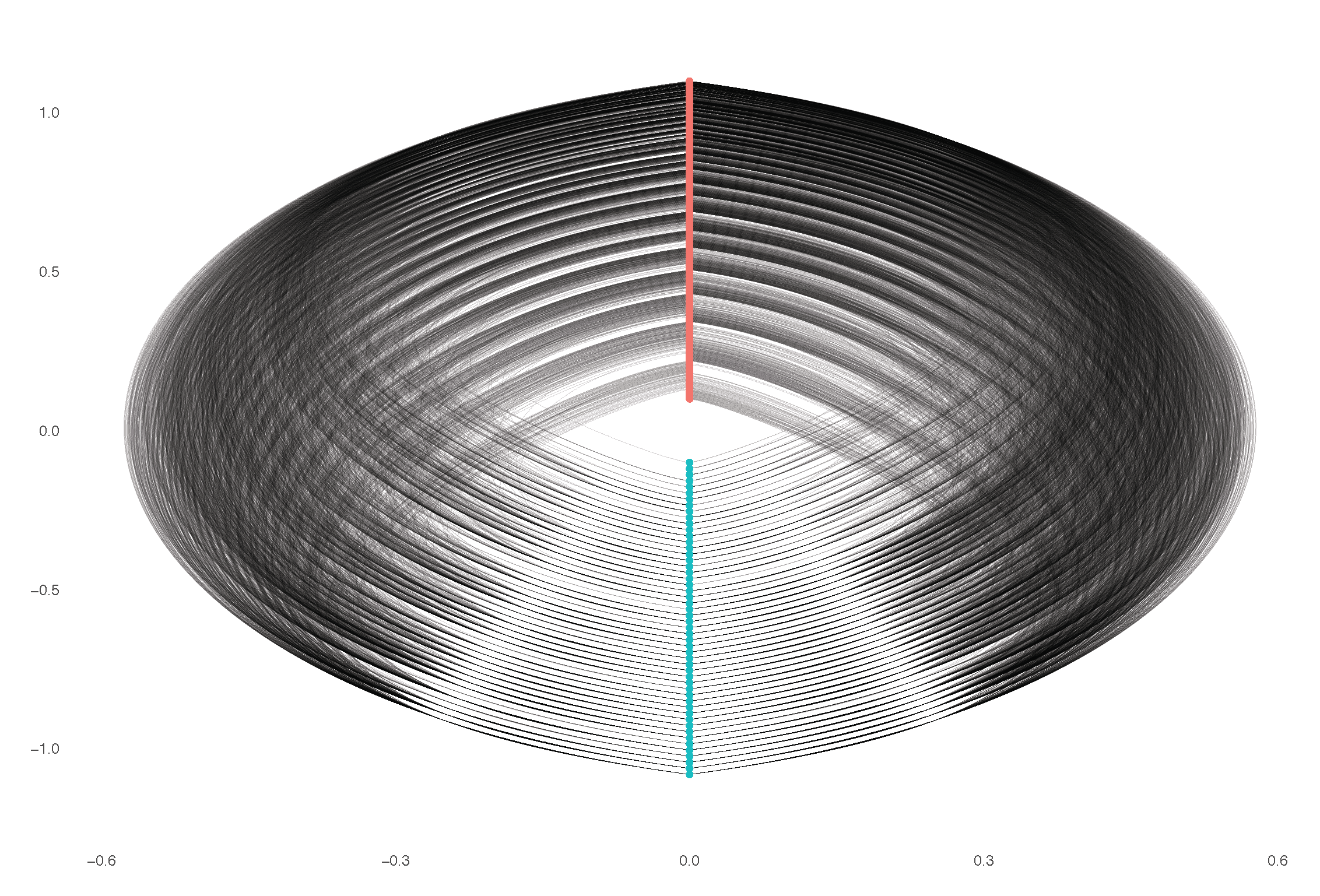

Supplement: Supplementary Figure 4 — Graph visualization of the drug-adverse drug reaction (ADR) network. A hive plot representation of the drug-ADR network is shown. This network comprises 52 drug nodes and 1,227 ADR nodes, with 9,597 links between them. Drugs nodes are arranged along the blue axis, while ADR nodes are arranged along the red axis. Edges between them are shown as Bezier curves. The transparency of each line is proportional to the edge strength. The network comprises a single large connected component containing all drugs and pathways. [file Image4.TIF]
